# Supplementary material for: A fault-intrusion-tolerant system and deadline-aware algorithm for scheduling scientific workflow in the cloud
Source: PeerJ Comput Sci. 2021 Nov 2;7:e747. doi: 10.7717/peerj-cs.747 (PMC8576554; doi:10.7717/peerj-cs.747)
Supplement: Supplemental Information 6 [file peerj-cs-07-747-s006.zip › Myproject1.1/docs/org/workflowsim/package-use.html]

Uses of Package org.workflowsim


JavaScript is disabled on your browser.


- Overview
- Package
- Class
- Use
- Tree
- Deprecated
- Index
- Help

- Prev
- Next

- Frames
- No Frames

- All Classes

# Uses of Package org.workflowsim

- Packages that use org.workflowsim

  | Package | Description |
  | --- | --- |
  |  |  |
  | --- | --- |
  | org.workflowsim |  |
  | org.workflowsim.clustering |  |
  | org.workflowsim.failure |  |
  | org.workflowsim.reclustering |  |
  | org.workflowsim.utils |  |
- Classes in org.workflowsim used by org.workflowsim

  Class and Description

  ClusteringEngine

  ClusteringEngine is an optional component of WorkflowSim and it merges tasks
  into jobs

  Job

  Job is an extention to Task.

  Task

  Task is an extention to Cloudlet in CloudSim.

  WorkflowEngine

  WorkflowEngine represents a engine acting on behalf of a user.

  WorkflowParser

  WorkflowParser parse a DAX into tasks so that WorkflowSim can manage them

  WorkflowScheduler

  WorkflowScheduler represents a scheduler acting on behalf of a user.
- Classes in org.workflowsim used by org.workflowsim.clustering

  Class and Description

  Job

  Job is an extention to Task.

  Task

  Task is an extention to Cloudlet in CloudSim.
- Classes in org.workflowsim used by org.workflowsim.failure

  Class and Description

  Job

  Job is an extention to Task.
- Classes in org.workflowsim used by org.workflowsim.reclustering

  Class and Description

  Job

  Job is an extention to Task.
- Classes in org.workflowsim used by org.workflowsim.utils

  Class and Description

  Job

  Job is an extention to Task.

- Overview
- Package
- Class
- Use
- Tree
- Deprecated
- Index
- Help

- Prev
- Next

- Frames
- No Frames

- All Classes
